# Macrophages in intestinal homeostasis and inflammation

**DOI:** 10.1111/imr.12192

**Published:** 2014-06-19

**Authors:** Calum C Bain, Allan McI Mowat

**Affiliations:** 1 Centre for Immunobiology, Institute of Infection, Immunity and Inflammation, University of GlasgowGlasgow, UK

**Keywords:** intestine, macrophages, monocytes, homeostasis, inflammation

## Abstract

The intestine contains the largest pool of macrophages in the body which are essential for maintaining mucosal homeostasis in the face of the microbiota and the constant need for epithelial renewal but are also important components of protective immunity and are involved in the pathology of inflammatory bowel disease (IBD). However, defining the biological roles of intestinal macrophages has been impeded by problems in defining the phenotype and origins of different populations of myeloid cells in the mucosa. Here, we discuss how multiple parameters can be used in combination to discriminate between functionally distinct myeloid cells and discuss the roles of macrophages during homeostasis and how these may change when inflammation ensues. We also discuss the evidence that intestinal macrophages do not fit the current paradigm that tissue-resident macrophages are derived from embryonic precursors that self-renew *in situ*, but require constant replenishment by blood monocytes. We describe our recent work demonstrating that classical monocytes constantly enter the intestinal mucosa and how the environment dictates their subsequent fate. We believe that understanding the factors that drive intestinal macrophage development in the steady state and how these may change in response to pathogens or inflammation could provide important insights into the treatment of IBD.

This article is part of a series of reviews covering Mucosal Immunity appearing in Volume 260 of *Immunological Reviews*.

## Introduction

As discussed elsewhere in this volume, the intestine is the largest mucosal surface of the body and is the single biggest compartment of the immune system. It is exposed constantly to an array of foreign antigens and must discriminate between harmful and harmless antigens to ensure that the appropriate response is mounted to each. Robust protective immune responses must be generated in response to pathogenic insult, but similar responses mounted against innocuous dietary proteins or commensal bacteria can lead to the development of chronic inflammatory disorders such as celiac disease and Crohn's disease, respectively.

Mononuclear phagocytes (MPs) comprising both macrophages (mϕ) and dendritic cells (DCs) are central to this discrimination process and therefore hold promise as targets for potential novel therapies to treat IBD. In this article, we discuss the role of mϕ in gut homeostasis and describe how they change when inflammation or infection is present. Furthermore, we discuss the ontogeny of intestinal mϕ and highlight that unlike many other tissue mϕ, those resident in the gut wall must be replenished continually by blood monocytes, whose fate is ultimately dictated by the environment into which they arrive. These unusual mϕ are ideally adapted to their surroundings and raise many questions about current views of the MP system and its functions.

## The challenges of identifying intestinal macrophages

MPs are one of the most abundant populations of leukocytes in the healthy intestinal mucosa, and this is the largest population of mϕ in the body 1,2. However, our understanding of the roles that individual subsets of DCs and macrophages play in maintaining homeostasis, and how they change when protective immune responses or inflammation occur has been stifled by the inability to identify these cells accurately. In large part, this is due to the substantial overlap in surface phenotype of intestinal DCs, mϕ, and other myeloid cells in the mucosa. For instance, although DCs in mice are traditionally identified by their co-expression of CD11c and major histocompatibility complex class II (MHCII), it is clear that mϕ of the gut wall express both these markers at high levels 3. Furthermore, CD11c has been shown to be expressed by plasma cells 4 and intestinal eosinophils in mice 5,6, with the latter also expressing the pan- mϕ marker F4/80 7,8. Expression of the fractalkine receptor CX3CR1 has also been used extensively to distinguish murine DCs from mϕ, with the proposal that these cells exhibited mutually exclusive expression of CD103, the α_E_ integrin, and CX3CR1, respectively 9–11. However, it is now clear that both mϕ and some DCs can express CX3CR1, albeit at different levels, while not all bona fide mucosal DCs are CD103^+^ 12–14. Thus, many of the surface markers regarded as ‘lineage-specific’ are now known to be expressed by multiple cell types, which in the past has led to the mischaracterization of mucosal MP. Some studies still use CD11c and MHCII co-expression alone to identify DC in non-lymphoid tissues, which makes the interpretation of these studies complex 15. Therefore, it is clear that unambiguous identification of intestinal mϕ and DC requires thorough multi-parameter analysis, with several surface markers needing to be examined in combination.

A useful approach to this comes from recent work that has demonstrated that expression of CD64 (the high affinity FcRγ1), F4/80 and MerTK 12,16,17, can be used in combination with CD11c and MHCII to define functionally distinct MP populations in mice. In the intestine, we have shown that CD11c^+^ MHCII^+^ MPs expressing CD64 and/or F4/80 are highly phagocytic cells with abundant foamy cytoplasm 12. These cells do not migrate to the mesenteric lymph nodes (MLNs) 13,14, cannot prime naive T cells 14, and their development *in vivo* requires the CSF-1R 18,19, but not the DC-specific growth factor flt3 ligand (flt3L) 12,13,16. Together, these features confirm the classification of CD64^+^ MPs as mϕ.

When identified appropriately, murine intestinal mϕ can be shown to express a number of characteristic surface molecules in addition to F4/80 and CD64, including the hemoglobin scavenger receptor CD163 and the mannose receptor (CD206) 12. Mature intestinal mϕ also express high levels of CX3CR1, although as discussed below, this appears to be acquired during local differentiation *in situ* and some cells of the mϕ lineage express lower levels of CX3CR1 12. It is important to note that CX3CR1 expression is not a characteristic shared by all tissue mϕ. For instance, lung alveolar mϕ, splenic red pulp mϕ, liver Kupffer cells, epidermal Langerhans cells, and mϕ of the peritoneal cavity all lack CX3CR1 expression 20, although fate mapping studies suggest they derive from a CX3CR1-expressing progenitor 20. Retention and/or upregulation of CX3CR1 expression only occurs in some mϕ. As well as those in the intestine, this includes microglia of the CNS and mϕ resident in the kidney 20. It seems likely that this is driven by signals from the environment (see below).

In contrast, CD64^−^ F4/80^−^ CD11c^+^ MHCII^+^ MPs display poor phagocytic activity, expand markedly in response to exogenous flt3L administration, and express the DC-specific transcription factor *Zbtb46* 12,13,21. As these CD64^−^ F4/80^−^ MPs also migrate constitutively to the MLNs in a CCR7-dependent manner and excel at priming naive T cells 13,14, they appear to represent classical migratory DCs. As discussed in more detail by Agace and colleagues elsewhere in this volume, it is important to mention that the intestinal DC compartment is itself heterogeneous, with discrete subsets identifiable on the basis of CD103 and CD11b expression 13,21,22. Although it was once thought that all mucosal DC expressed CD103, it is now clear that bona fide CD103^−^ DCs exist 13,21 and that CD103 cannot be used as a *de facto* marker of mucosal DCs. Importantly, CD103^−^ CD11b^+^ DCs also express intermediate levels of CX3CR1, making them almost identical to mϕ in terms of their surface phenotype. This emphasizes the need to use multiple markers to distinguish intestinal DCs from mϕ, using markers such as CD64, as well as CD26 and CD272, recently identified by the Immunological Genome Project as DC-specific markers 23.

Characterization of mϕ in the human gut can also prove difficult, with investigators using different panels of pan-myeloid cell markers, including CD33, CD14, and CD13 24–26. Interestingly, more powerful multi-parameter analysis has shown recently that there is conservation of surface marker expression between mouse and human which may prove useful. For instance, expression of CD64 also appears to allow accurate identification of mϕ in human gut and, like those found in the mouse gut, they express high levels of MHCII, CD163, and CD68 12,26,27. Unlike murine intestinal mϕ, those in the human mucosa essentially lack CD11c expression, except for a small population that also expresses high levels of the LPS co-receptor CD14, normally found at low levels on mature macrophages 12. As discussed below, these CD11c^+^ CD14^hi^ cells may represent recently arrived monocytes from blood. Importantly, CX3CR1 has also been shown to be expressed by intestinal macrophages in humans 28, although it is unclear whether levels of CX3CR1 expression identify different subsets of MP in man.

It has become commonplace to classify mϕ into one of two major subtypes based on their surface receptor expression and which mediators they produce – M1 and M2 29. However, gut-resident mϕ do not fit readily into this ‘M1-M2 paradigm’, having some of hallmarks of both M1 and M2 mϕ. For instance, they express high levels of MHCII and produce TNFα constitutively 12,30, features normally associated with M1 or ‘classically activated’ mϕ 31. However as discussed above, they also express CD206, CD163, and produce interleukin-10 (IL-10), features associated with M2 or M2-like mϕ 32. However, they fail to express arginase which is a cardinal feature of M2 mϕ 12. Thus, like most tissue mϕ *in vivo*, those resident in the gut wall adapt to their local environment in complex and specific ways that may not be reflected by the rigid classification of the M1-M2 paradigm.

## Anatomical distribution of intestinal macrophages

Mϕ are found in the mucosa throughout the entire GI tract and are located mostly in the *lamina propria* (LP) in close proximity to the epithelial monolayer 2. A discrete population is also present in the smooth muscle layers of the gut wall, playing important roles in regulating intestinal motility 33,34. The number of mϕ in the LP varies between the different parts of the GI tract, with more being found in the colon than the small intestine of humans and rodents (35,36, authors’ unpublished observations). Although it has been reported that there is a continuous gradient in mϕ numbers from proximal to distal ends of the entire mouse intestine 36, this may not be the case in humans, where the colon appears to have similar numbers of macrophages throughout its length 35.

## Functions of macrophages in the steady state mucosa

**Figure 1:**
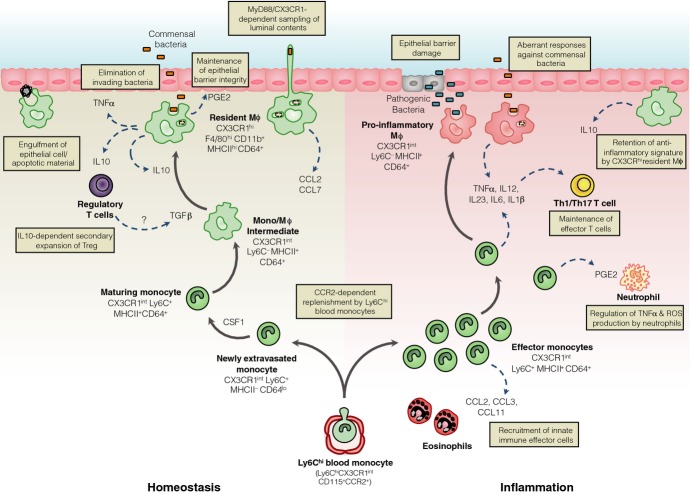
Under steady state conditions, Ly6C^hi^ monocytes constitutively enter the intestinal mucosa and differentiate into mature CX3CR1^hi^ F4/80^+^ macrophages (mϕ) through a series of short-lived CX3CR1^int^ intermediaries. These CX3CR1^hi^ mϕ are positioned immediately beneath the epithelial barrier and contribute to its integrity by secreting PGE2 which stimulates proliferation of epithelial progenitors. This positioning coupled with their high phagocytic capacity means they are poised to capture and destroy any invading commensals or pathogens, as well as clearing apoptotic or senescent cells. They may also be able to directly sample the luminal contents by the extending processes between the cells of the intestinal epithelial barrier. CX3CR1^hi^ mϕ also produce interleukin-10 (IL-10) constitutively, which facilitates secondary expansion of regulatory T cells in the mucosa and may also condition newly arrived monocytes. Regulatory T cells may also contribute to the conditioning of newly extravasated monocytes through their production of TGFβ. When homeostasis is perturbed by inflammation or infection, Ly6C^hi^ monocytes and their CX3CR1^int^ derivatives accumulate in large numbers and display enhanced pro-inflammatory characteristics. They produce pro-inflammatory cytokines which may support the maintenance of other effector cells such as IFNγ producing and IFNγ^+^ IL-17 double producing T cells. They also orchestrate the recruitment of other innate effector cells such as eosinophils through secretion of inflammatory chemokines. Importantly, during inflammation, CX3CR1^hi^ mϕ retain their anti-inflammatory signature, e.g. IL-10 production. Elicited classical monocytes may also play a regulatory role by controlling the production of TNFα and ROS by neutrophils.

Like most tissue mϕ, those resident in the normal gut wall play essential housekeeping functions, such as the clearance of apoptotic or senescent cells, and tissue remodeling 35,37,38 (*Fig. *1). This would be consistent with their expression of scavenger receptors such as CD36 that can bind and engulf apoptotic cells 39. Furthermore, intestinal mϕ are known to produce a variety of cytokines and other soluble factors that help maintain tissue homeostasis. One such factor is PGE2 40, which allows local mϕ to stimulate the proliferation of epithelial progenitors in intestinal crypts 41, thereby regulating the integrity of the epithelial barrier. Reflecting the importance of these homeostatic functions, selective depletion of mϕ by targeting the CSF1R leads to enhanced susceptibility to dextran sodium sulfate (DSS)-induced colitis 42 and epithelial repair in this model requires MyD88 signaling in myeloid cells 43.

However, it is in their role as innate immune effector cells that mϕ are best known. Resident intestinal mϕ are highly phagocytic and are actively bactericidal 44, which together with their positioning immediately under the epithelial monolayer, means they are ideally located to capture and destroy any material breaching the epithelial barrier (*Fig. *1). They also express high levels of the TREM2 phagocytic receptor 30, which enhances their ability to engulf bacteria 45. However, one of the most characteristic features that distinguishes intestinal mϕ from their counterparts elsewhere in the body is that exposure to bacteria or their products does not trigger pro-inflammatory responses by mucosal mϕ 12,44. Similarly, ingestion of bacteria does not stimulate respiratory burst activity 46 or the generation of nitric oxide 47 by intestinal mϕ. There is also no enhanced production of TNFα, IL-1, IL-6, or other inflammatory cytokines and chemokines in response to ligation of TLR or intracellular NOD receptors 44,48. However, recent studies in mice indicate that intestinal mϕ are not completely anergic in terms of cytokine production, as they show constitutive production of substantial amounts of IL-10, as well as low levels of TNFα 12. Although it may seem surprising that mϕ constitutively produce this prototypical pro-inflammatory cytokine, TNFα may have much wider effects. For instance, TNFα can regulate enterocyte growth and alter the permeability of the epithelial barrier 49. Furthermore, TNFα stimulates production of matrix metalloproteinases and other tissue remodeling enzymes in the intestinal mesenchymal cells that are central to regulating epithelial cell function 50.

Given their high expression of MHCII and their ability to take up orally administered protein antigens 51–53 and bacteria 37, intestinal mϕ may also serve as antigen-presenting cells, interacting with and influencing the differentiation of CD4^+^ T cells (*Fig. *1). It has also been suggested that mϕ may play a selective role in initiating the differentiation of FoxP3^+^ regulatory T cells in the intestine 54. However, as mϕ do not migrate to the MLN 14 and the intestinal mucosa is essentially devoid of naive CD4^+^ T cells 55, they are unlikely to be involved in the initial priming of naive CD4^+^ T cells. Furthermore, LP mϕ are extremely poor at activating naive CD4^+^ T cells *in vitro* 14. Thus if mucosal mϕ do present antigens to CD4^+^ T cells, it seems more likely that this involves lymphocytes that have returned to the mucosa after being first primed in the MLN. Consistent with this idea, Hadis *et al*. 56 have recently shown that mϕ facilitate the secondary expansion and maintenance of antigen-specific FoxP3^+^ Treg in the intestinal mucosa through their production of IL-10 (*Fig. *1). The importance of this local fine tuning of T-cell responses by mϕ is highlighted by the loss of FoxP3 expression and suppressive capacity that occurs when T cells are rendered unresponsive to IL-10 through deletion of the IL-10R 57. Further evidence that intestinal mϕ may contribute to the generation of homeostatic Treg comes from work showing that mice lacking TRAF6 expression in CD11c^+^ MP develop a spontaneous Th2 cell-mediated enteritis. This is associated with a defective ability of mucosal MP to induce FoxP3^+^ T cells *in vitro* and reduced numbers of Treg in the LP 58. Although interpreted as reflecting a role for ‘DCs’, the relevant myeloid cells were defined only as CD11c^+^ and as we have noted, most of these cells in the normal LP are in fact mϕ. Further work is needed to define the precise cellular basis of TRAF6-mediated generation of Treg in the LP.

Intestinal mϕ may also contribute to the maintenance of other T cells in the mucosa. For instance, the production of IL1β in response to TLR stimulation may be relatively preserved in intestinal mϕ and this has been reported to help support the development of Th17 cells in the steady state intestine 59. It remains to be determined whether these effects of intestinal mϕ on T cells require cognate interactions via peptide-MHC complexes and the TCR, or whether it involves non-specific factors such as polarizing cytokines alone, as has been suggested for the generation of Th17 responses by mucosal CD103^+^ CD11b^+^ DCs 60.

Recent studies have proposed a further way in which mucosal mϕ may contribute to indirectly to T-cell priming, through Connexin 43-mediated transfer of soluble antigen obtained from the lumen to neighboring CD103^+^ DCs 52. This cooperative process has been suggested to be important for the development of oral tolerance and if confirmed, these findings could indicate an important new role for mϕ in the steady state intestine.

Exactly how mϕ in the LP acquire luminal antigen remains to be established with certainty. One possibility is that they may extend transepithelial dendrites (TED) across the epithelium to capture soluble antigen in the lumen (*Fig. *1). Originally thought to be derived from DCs on the basis of the expression of MHCII and CD11c 61,62, it now seems clear that these TED originate from CX3CR1^+^ mϕ. Their formation requires CX3CR1 61,63 and indeed, the induction of oral tolerance to protein antigens is impaired in CX3CR1-deficient mice 52,56, consistent with a requirement for CX3CR1-mediated uptake of luminal antigen in this process. However, the physiological significance of such TED remains unclear. Not only did the original reports disagree on which part of the small intestine they were present in and if they were dependent on TLR signaling 61,62, but more recent studies have failed to find TED anywhere in the steady state intestine 64. Furthermore, earlier studies on the uptake of soluble protein from the intestinal lumen reported that this process was not dependent on the CX3CL1–CX3CR1 axis 14. Finally, as we have noted, other work has also proposed that the defect in oral tolerance in CX3CR1-deficient mice reflects an absence of IL-10 production by intestinal mϕ 56. If and how LP mϕ contribute to the uptake of soluble antigen and then help shape subsequent immune responses clearly warrants further investigation.

Despite their phagocytic activity and morphological appearance, there is similar uncertainty about whether LP mϕ play a role in the capture of and induction of immunity or tolerance to local bacteria. Although it is well established that CD103^+^ DCs can present bacterial antigen to naive T cells in the MLN 53, initial reports indicated that the TED formed by MP in the mucosa could capture *Salmonella* organisms in the lumen 61,62. Furthermore, a very recent study showing that CX3CR1^+^ cells may transport *Salmonella* in lymph to the MLN was interpreted as showing a role for migratory mϕ in this process 65. However, this phenomenon was only revealed after depletion of the normal microbiota with antibiotics and the study did not exclude the possibility that the antigen loaded CX3CR1^+^ MP belonged to the population of bona fide CX3CR1 expressing DC that we and others have observed in intestinal lymph 13,14. Again, these findings reveal how much is still to be understood about how mϕ contribute to the induction and shaping of mucosal immune responses.

## Ontogeny of intestinal macrophages in the steady state

### The mononuclear phagocyte system

Tissue mϕ are traditionally viewed as part of the mononuclear phagocyte system (MPS). First described in 1972 66, the concept of the MPS proposes that all tissue mϕ are the terminally differentiated progeny of blood monocytes, which constitutively enter tissues under steady state conditions, and are themselves replaced by rapidly dividing precursors in the bone marrow (BM). Originating from the common myeloid progenitor, which also generates DCs and granulocytes, a common macrophage and dendritic cell progenitor gives rise to both committed DC precursors (pre-DCs) and to monocytes 67,68, with the latter passing through a recently identified common monocyte progenitor 69. The development of monocytes via these processes is dependent on the transcription factor PU.1 and the growth factors CSF1 (M-CSF), CSF2 (GM-CSF), and IL-3 69.

It is now known that the monocyte pool is heterogeneous and, in mice, CSF1R^+^ (CD115) monocyte subsets can be distinguished by their expression of Ly6C/Gr1 and the chemokine receptors CCR2 and CX3CR1 70. The majority of blood monocytes expresses high levels of Ly6C/Gr-1, CCR2 and CD62L, but low levels of CX3CR1 (so-called Ly6C^hi^ monocytes). The entry of Ly6C^hi^ monocytes into the circulation from the BM is dependent on the CCL2-CCR2 chemokine axis and as a result, mice with genetic deletion of either of these molecules have markedly fewer circulating monocytes 71. Although initially referred to as ‘inflammatory’ monocytes because of an apparent preference to migrate to inflamed tissues, these Ly6C^hi^ monocytes are now classified as ‘classical’ monocytes, as they fulfill all the roles previously attributed to generic monocytes in the MPS. In human, an analogous population of classical monocytes is identified as CD14^hi^ CD16^−^ and these also express CCR2 72.

A smaller population of murine monocytes expressing lower levels of Ly6C and CCR2, but higher amounts of CX3CR1 (Ly6C^lo^ monocytes) were initially proposed to be the precursors of steady state tissue mϕ 70. However, little direct evidence exists to support this idea, and more recently it has become clear that the primary function of Ly6C^lo^ monocytes is to act as phagocytes in the bloodstream, patrolling and maintaining the vasculature 73,74. As such, they are now commonly referred to as ‘patrolling’ or non-classical monocytes 75, and there is accumulating evidence that there is a developmental relationship between classical and non-classical blood monocytes, with the latter being the CSF1R-dependent progeny of the former 20,76. The equivalents of these non-classical Ly6C^lo^ monocytes in human are identified as CD14^lo^ CD16^+^, and these express relatively high levels of MHCII 70. Whether they exert similar patrolling functions to their murine counterparts is not known, although gene expression analysis has revealed many parallels between the monocyte subsets in mouse and human 72.

### Macrophage development independent of the MPS

The validity of the MPS has been questioned recently, with multiple reports from independent groups proposing that the majority of tissue mϕ exists independently of blood monocytes 77–79. Using a variety of techniques including fate mapping, parabiosis, and analysis of proliferative capacity, it has been shown that epidermal Langerhans cells 79,80, lung alveolar mϕ 20,81, liver Kupffer cells 20,77, microglia of the CNS 20,77,82,83, and mϕ of the peritoneal cavity 78 derive from embryonic precursors that seed tissues early in development and maintain themselves through *in situ* proliferation. Although there is debate about whether these precursors originate in the mesenchyme of the yolk sac, or from the fetal liver, an assumption has come about that all tissue mϕ populations may be derived from these early progenitors 84.

Our recent work shows that the intestine is an exception to this paradigm, as mϕ in the adult steady state mucosa require continuous replenishment by Ly6C^hi^ blood monocytes 12. Although earlier studies had shown that Ly6C^hi^ monocytes could enter the colonic mucosa following intense depletion of resident MP 9, it was unclear whether such a mechanism operated in the unmanipulated mucosa and if the progeny represented mϕ or DCs 10,85. Using an adoptive transfer approach, we could demonstrate that Ly6C^hi^ monocytes constantly enter the normal colonic mucosa and mature into CD64^+^ F4/80^hi^ CX3CR1^hi^ MHCII^+^ cells 12,16, indistinguishable from endogenous resident mϕ (*Fig. *1). The development of mature mϕ involves a local differentiation process, in which a series of intermediaries lose Ly6C and upregulate F4/80, CX3CR1, CD163, and CD206, as well as acquiring CD11c and MHCII expression 12, features that account for their previous misclassification as DCs 9,10. Full maturation appears to take around 5 days and as well as adopting the phenotypic signature of mature colonic mϕ, the differentiating monocytes begin to acquire the characteristic functional signature of intestinal mϕ. Thus they become increasingly phagocytic and begin to produce IL-10, as well as developing resistance to TLR stimulation 12.

There are multiple lines of evidence to support the idea that monocyte recruitment is the principal mechanism of replenishment of intestinal mϕ. Firstly, mϕ in the adult mucosa have very poor proliferative capacity 12,86, suggesting *in situ* self-renewal plays little or no role under normal physiological conditions. Secondly, mice in which the CCL2–CCR2 axis has been disrupted by genetic deletion of either molecule have significantly fewer colonic mϕ 12,87. Next, the intestinal mϕ pool derives almost exclusively from WT BM in WT:CCR2^−/−^ mixed BM chimeric mice 16. Finally, cells matching the phenotype of intestinal mϕ are completely eliminated by DT-mediated depletion in CCR2-DTR mice 88. This requirement for the CCL2-CCR2 axis does not simply reflect its role in BM egress of monocytes, as CCR2-sufficient Ly6C^hi^ monocytes fail to enter the CCL2-deficient gut mucosa in WT-CCL2^−/−^ parabiotic mice 87, demonstrating that the CCL2-CCR2 axis is needed for entry of monocytes into the intestinal LP. Although the source of CCL2 in the intestine is unclear, CX3CR1^hi^ resident mϕ in the normal mucosa express high levels of the CCR2 ligands CCL2 and CCL7 87,89 and therefore they may control their own replenishment. This mechanism has been shown to occur in the mouse uterus, where CSF1R-signaling results in production of CCR2 ligands by uterine mϕ 90. Intestinal mϕ are all labeled in MacGreen mice in which GFP is expressed under control of the CSF1R promoter 91,92 and thus it is intriguing to speculate that rather than being required for their local proliferation/survival, CSF1R-signalling in gut mϕ may be required to elicit monocyte recruitment to the mucosa, as has been suggested in other tissues 90,93.

As CCR2- and CCL2-deficient mice are not completely devoid of intestinal mϕ 12,87, there may be other mechanisms of replenishment in addition to the CCL2-CCR2 axis. This is unlikely to involve non-classical Ly6C^lo^ monocytes, as these fail to migrate into non-lymphoid tissues when adoptively transferred 9,12, and as discussed above, they seem to have a distinct effector role patrolling the vasculature 73. In addition, although CX3CR1-deficient mice have markedly fewer circulating Ly6C^lo^ monocytes due to impaired survival 63,94, the intestinal mϕ compartment of these mice remains unaffected in size 56. *In vitro* studies have also implicated TGFβ and IL-8 in the recruitment of monocytes to the intestinal mucosa 27,86, but the role of these and other mediators has not been addressed *in vivo*.

Why the gut mucosa should need continuous recruitment and differentiation of Ly6C^hi^ monocytes is not completely understood. An obvious possibility is that this is required to monitor the constant exposure to the commensal microbiota and other environmental agents found at barrier surfaces. Although the abundance of differentiating monocytes is reduced in the dermis of germ free (GF) mice 16, different studies mice have reached conflicting conclusions on the size of colonic mϕ compartment in GF mice 11,95,96. Furthermore, whether GF conditions or antibiotic treatment alter monocyte recruitment to the gut mucosa and/or their subsequent differentiation has never been formally examined.

An analogous monocyte differentiation process seems to exist in human intestine. We recently showed that CD14^hi^ monocytes appear to differentiate into mature mϕ through a series of intermediaries which involves downregulation of CD14 and CD11c, and upregulation of MHCII, CD209 and CD163 12. Together these findings indicate that classical monocytes are the principal source of the resident intestinal mϕ pool in mouse and human.

## Monocyte education in the mucosa

The environmental cues that shape monocyte differentiation in the intestinal mucosa remain to be identified with certainty, and it is likely that multiple factors are responsible for the imprinting of the phenotypic and functional characteristics adopted by maturing monocytes (*Fig. *1). These factors are likely to be specific to the intestinal mucosa, as monocytes do not acquire the same characteristics after entering other mucosal sites such as the lung 97. Multiple growth factors are responsible for the maturation of cells of the MPS, including flt3L, CSF1, and CSF2. As noted above, intestinal mϕ do not expand in response to exogenous flt3L and remain equally abundant in flt3L-deficient mice (16, authors’ unpublished data). Similarly, although some mϕ such as lung alveolar mϕ have been shown to depend on the CSF2R for their development 78,98, it is unlikely that this applies to intestinal mϕ, as cells matching their phenotype are derived equally from CSF2R-deficient BM in WT:CSF2R-deficient BM chimeras 99. The development of intestinal mϕ requires signaling through the CSF1R and as a result, they are derived almost exclusively from WT BM in WT:CSF1R^−/−^ mixed BM chimeric mice 19. There are currently two identified ligands of the CSF1R: CSF1 and IL-34. To date, only microglia and Langerhans cells of the epidermis have been reported to depend on IL-34 and in these cases, this appears to be the driver of *in situ* self-renewal 100,101, making it seem unlikely that IL-34 will play a role in intestinal mϕ homeostasis. However this has never been examined directly. It is likely that CSF1 is the predominant ligand required in the gut as evidenced by a marked reduction in intestinal mϕ in osteopetrotic *Csf1*^*op/op*^ mice, which have a mutation in the gene encoding CSF1 102. It also remains to be determined at which stage of intestinal mϕ development CSF1R signalling is involved and specifically whether it is important simply for the generation of progenitors, or also for the differentiation of monocytes that enter the mucosa. The loss of Ly6C and upregulation of F4/80 that occurs during the transition of Ly6C^hi^ to Ly6C^lo^ monocytes in blood is dependent on CSF1-CSF1R interactions 18,20, as is the development of monocyte-derived mϕ in other tissues 18,19. However, it seems unlikely that CSF1R signalling alone can account for all aspects of intestinal mϕ development given the fact that adoptively transferred monocytes generate entirely distinctive mϕ in this tissue. Thus, the local environment of the intestinal mucosa must play the definitive role in local mϕ development.

A distinctive feature of intestinal mϕ is their expression of MHCII at levels even greater than those seen on mucosal DCs. Indeed, acquisition of MHCII is one of the first phenotypic changes to occur during monocyte maturation in the intestine, although its functional significance remains unclear 12. This does not appear to be a default property of monocyte maturation, as it is not a universal feature of other monocyte-derived tissue mϕ 90,103 or of the blood Ly6C^lo^ monocytes, which mature from Ly6C^hi^ monocytes 97. IFNγR signaling is known to induce MHCII on mϕ 104, but MHCII expression by intestinal mϕ is normal in IFNγR-deficient mice (our unpublished observations). It also appears to be independent of CSF2, IL-10, and lymphocytes or their products (our unpublished observations). Recently, the Randolph laboratory has suggested that signals from vascular endothelial cells may instruct upregulation of MHCII by extravasating monocytes 97. However, whether this occurs as monocytes enter the gut mucosa remains unclear.

As noted above, acquisition of the CX3CR1^hi^ phenotype appears to be associated with the development of an anti-inflammatory signature in fully differentiated intestinal mϕ. Given that the majority of mϕ in other tissues lack CX3CR1, it is clear that the local environment of the gut must instruct developing monocytes to upregulate this molecule. The factor responsible has not been identified, although TGFβ is a clear candidate, as it is known to induce CX3CR1 expression by microglia in the brain 105. There is accumulating evidence that CX3CR1 itself may play a role in controlling the differentiation and function of intestinal mϕ. Firstly, as discussed above, the CX3CL1–CX3CR1 axis has been shown to control the ability of CX3CR1^+^ MPs to extend cellular protrusions into the intestinal lumen 61,63,106. Although this remains an area of contention, it is conceivable that the expression of CX3CL1 by enterocytes 63 could influence the anatomical positioning of mϕ under the epithelial layer. However to date, it has not been reported that CX3CR1-deficient mϕ inhabit different anatomical locales than their WT counterparts. Next, the CX3CL1–CX3CR1 axis has been shown to be required for optimal production of IL-10 by intestinal mϕ 56 and finally, CX3CR1-deficiency has been reported to lead to decreased numbers of intestinal mϕ 107,108 as well as altering susceptibility to chemically induced colitis. However, given that CX3CR1-deficient mice have been shown both to be protected 11,63 and more susceptible to experimental colitis 107, further investigation of the CX3CR1–CX3CL1 axis is required to elucidate its involvement in the control of mϕ function in the intestine.

The IL-10–IL-10R axis plays a crucial role in conditioning the behavior of mϕ in the mucosa. One such effect is the upregulation of scavenger receptors such as CD206 and CD163, which is reduced in IL-10-deficient mice 96,109. Even more marked is the requirement for IL-10 in the hyporesponsiveness of intestinal mϕ to pro-inflammatory stimuli 44 (see below). The cellular source of the IL-10 remains unclear. Although mϕ themselves produce IL-10 constitutively and this could act in an autocrine manner, selective knockout of IL-10 in LysM^+^ cells appears not to lead to spontaneous IBD in mice 110, suggesting other cells are more important. This is likely to be IL-10-producing FoxP3^+^ CD4^+^ T cells, as these are abundant in the normal intestine and IBD occurs after specific deletion of IL-10 in these cells 111,112. The induction of IL-10 in maturing mϕ may be a response to colonization of the intestinal tract by the commensal microbiota, as colonic mϕ from mice reared in GF conditions produce less IL-10 95,96. However given that this failure to upregulate IL-10 production cannot be recapitulated in MyD88^−/−^ mice 95, the mechanisms underlying this phenomenon require further investigation. Interestingly, the production of protective IL-10 by CD4^+^ T cells is also dependent on the microbiota 113. While it has been reported that IL-10 may be important for more general aspects of maturation of newly recruited monocytes in the peritoneum, including upregulation of MHCII 114, our work on inflamed intestine suggests that the lack of MHCII may reflect the failure of monocytes to differentiate fully due to the enhanced inflammation found in the absence of IL-10, rather than a direct effect of IL-10 (see below).

## Mechanisms of inflammatory anergy in intestinal macrophages

Hyporesponsiveness to activation via TLRs and other stimuli is a cardinal feature of resident intestinal mϕ. Early work suggested that this was the result of a failure to express pattern recognition receptors. However, it is now clear that gut-resident mϕ in mice and human express a full range of TLR 12,27, and it is now believed that regulation of adapter molecules downstream of the TLR may instead be responsible for the hyporesponsiveness. For instance, signaling molecules such as CD14, MyD88, TRAF-6, MD2, TRIF, and IRAK1 appear to be downregulated in mature intestinal mϕ 27,44,89. In parallel, mechanisms that inhibit TLR signaling and/or NF-κB activation (e.g. IRAK-M and IkBNS) appear to overexpressed in gut-resident mϕ 27,89,109, suggesting that molecules that propagate TLR signaling are targeted rather than the TLR expression itself.

At least some of these inhibitory processes may be driven by IL-10 109. In mice, the hyporesponsiveness develops progressively as monocytes mature in the mucosa and it correlates with increasing IL-10 production 12. As a result, colonic mϕ from IL-10^−/−^ mice or mice with LysM-mediated deletion of the IL-10R signaling molecule STAT3 display exaggerated pro-inflammatory responses to bacterial products 95,96,109,115, and the animals develop spontaneous colitis 116,117. Importantly, IL-10R deficiency is also associated with an early onset form of severe IBD in human 118,119. Thus, it is clear that IL-10 is one of the most important factors ensuring mϕ quiescence in the face of environmental stimuli. However, other mechanisms are likely to be involved. For instance, TGFβ has been shown to render blood monocytes hyporesponsive to a variety TLR ligands 27 and active TGFβ is abundant in the steady state LP 120. The spontaneous, microbiota-dependent enteritis that occurs in mice lacking expression of TRAF6 in CD11c^+^ MP could also reflect defective TGFβR-dependent signaling in mϕ, as TRAF6 and its downstream partner TAK1 are critical components in this pathway 58.

The control of mϕ activity may also involve dedicated inhibitory receptors including SIRPα and CD200R1. Indeed, the CD200–CD200R1 axis appears to play an important role in regulating alveolar mϕ activity 121. However, our recent work suggests that the same regulatory loop is dispensable in the intestinal mucosa, with CD200R1-deficient colonic mϕ showing no signs of dysregulation 8. Furthermore, deletion of CD200R1 or its only ligand, CD200 does not render mice more susceptible to DSS-induced colitis, as would be expected if mϕ activity was not controlled appropriately 8. Although resident mϕ express high levels of SIRPα, whether this plays any role in controlling intestinal mϕ activity through interaction with its ligand CD47 has never been formally tested.

The development of the hyporesponsive state appears to involve recognition of microbial signals, as colonic MP from GF mice respond robustly to stimulation with the TLR4 agonist LPS 96. However, it must be noted that in this study mϕ were identified only as CD11b^+^ CD11c^−^ cells, which will include many cell types and exclude the majority of resident mϕ which express CD11c. Therefore, the full impact of the microbiota on the behavior of resident colonic mϕ remains to be elucidated.

Together, these findings demonstrate that fully responsive Ly6C^hi^ monocytes continuously enter the steady state intestinal mucosa as a surveillance measure. In the absence of any threat, the monocytes adopt an anti-inflammatory phenotype which is imprinted by local factors in the mucosa. The nature and mechanisms of action of these mediators remain to be defined fully.

## Monocytes and macrophages in intestinal inflammation

It is well known that the composition of the human intestinal mϕ pool changes considerably when there is perturbation of homeostasis. For instance, in the mucosa of Crohn's disease and ulcerative colitis patients, there is an accumulation of pro-inflammatory macrophages. These can be identified by their high expression of CD14, in contrast to the CD14^lo/−^ population of resident mϕ 12,24,122,123. The ability of these cells to produce large amounts of mediators such as IL1, IL6, TNFα, reactive oxygen intermediaries and nitric oxide makes them quite distinct from the mϕ found in healthy intestine and has led to interest in the idea of targeting the monocyte-macrophage lineage for therapeutic purposes. For these reasons, it has become important to establish whether the inflammatory cells represent newly arrived monocyte-derived cells, or are resident cells that have altered their behavior in the presence of inflammation.

## Mouse models of intestinal inflammation

There is similar infiltration of pro-inflammatory monocytes and mϕ in animal models of intestinal inflammation such as colitis induced by administration of DSS 12,89, by the adoptive transfer of naive CD4^+^ T cells into lymphopenic hosts 16,30, or after infection with *Helicobacter hepaticus* coupled with neutralization of IL-10 (our unpublished observations). In all these cases, the infiltrate is characterized by a reversal in the ratio of CX3CR1^hi^ and CX3CR1^int^ cells, caused by the accumulation of Ly6C^hi^ monocytes and their CX3CR1^int^ progeny (*Fig. *1). The infiltrating CX3CR1^int^ cells display typical pro-inflammatory characteristics, including the production of TNFα, IL-6, IL-1β, IL-12, IL-23, and expression of iNOS. In contrast, the remaining CX3CR1^hi^ mϕ retain their anti-inflammatory signature, continuing to produce high levels of IL-10 and being TLR hyporesponsive, suggesting that resident mϕ do not turn rogue during inflammation 12,16,30,89. Not unexpectedly, CCR2 is required for the entry of pro-inflammatory cells into the inflamed colonic mucosa, with considerably fewer CCR2^−/−^ cells entering the mucosa when co-transferred with their WT counterparts 89,124. A pathological role for these elicited Ly6C^hi^ monocytes is evidenced by amelioration of colitis in monocytopenic CCR2-deficient mice 124 and in mice depleted of CCR2-expressing cells 89. Furthermore, TNFα production by these cells appears to be central to disease progression, as the severity of DSS-induced colitis is reduced in mice in which Ly6C^hi^ monocytes are deficient in TNFα production 9. On the basis of our studies of DSS colitis, we have proposed that the normal monocyte differentiation process is arrested during inflammation, resulting in the retention of cells that remain responsive to TLR stimulation and produce copious amounts of pro-inflammatory cytokines and chemokines 12,30,89. Why these processes are disrupted in inflammation remains to be determined, but presumably reflects alterations in the local microenvironment, leading to defects in the conditioning mechanisms that normally specify maturation of monocytes into anti-inflammatory mϕ. Clearly however, the resident mϕ themselves cannot be influenced by these changes, indicating these are likely to be terminally differentiated cells and raising the possibility that homeostasis may be restored relatively quickly if the inflammatory environment can be modified.

Although Ly6C^hi^ monocytes and their immediate progeny appear to be detrimental during sterile models of intestinal inflammation 12,124, they are also indispensable for the eradication of certain enteric pathogens. This is consistent with findings that elicited CX3CR1^int^ cells, many of which are likely to be derivatives of Ly6C^hi^ monocytes, have been shown to take up *Salmonella* organisms as efficiently as resident CX3CR1^hi^ mϕ 37. During the acute ileitis caused by oral inoculation of certain mouse strains with the protozoan parasite *Toxoplasma gondii*, there is accumulation of Ly6C^hi^ monocytes, which display the same behavior as those arriving into the DSS-inflamed colon, including the production of the pro-inflammatory cytokines TNFα and IL-12, and reactive nitrogen species 125. The protective role of these monocytes is demonstrated by the fact that both CCR2- and CCL2-deficient mice succumb to lethal toxoplasmosis, and that the adoptive transfer of CCR2-competent Ly6C^hi^ monocytes can rescue this lethality 125. Interestingly, the CCL3–CCR1 chemokine axis driven by innate lymphoid cells is also involved in the recruitment of pro-inflammatory Ly6C^hi^ monocytes and protective immunity in this model of infection, indicating that CCR2 may not be the only mechanism responsible for accumulation of monocytes during inflammation 126. However, no defects in steady state intestinal mϕ have been reported in mice lacking CCR1, perhaps suggesting distinct roles for individual chemokine receptors under different conditions.

Ly6C^hi^ monocytes have also been implicated in the protective immune response to *Citrobacter rodentium*, a mouse model of enteropathogenic and enterohaemorrhagic *E. coli* infection in human, with delayed clearance in CCL2- and CCR2-deficient mice 127. However, recent elegant work has questioned this conclusion, by showing that ablation of the entire monocyte-macrophage compartment in mice with LysM-driven expression of DTR by CSF1R-expressing cells has no effect on susceptibility to *C. rodentium* infection 128. In contrast, the robust Th17 response needed to clear this organism 127 is absolutely dependent on classical migratory DC expressing zbtb46 21. Interestingly however, monocyte-derived macrophages may make an additional contribution to the adaptive immune response under these conditions, by producing IL12, which maintains IFNγ^+^ and IFNγ^+^ IL17^+^ effector T cells in the mucosa 128. Thus, whereas resident mϕ maintain regulatory T cells via IL-10 production, recently elicited monocytes may maintain effector T cells through the production of pro-inflammatory cytokines (*Fig. *1).

Contrasting with their role as inflammatory effector cells, recent studies suggest that Ly6C^hi^ monocytes may have a wider ability to regulate other aspects of intestinal immunity (*Fig. *1). For instance, in the DSS colitis model, Ly6C^hi^ monocytes mediate the recruitment of eosinophils through their production of CCL11 (eotaxin) 129. In *T. gondii*-induced ileitis, elicited Ly6C^hi^ monocytes may actually prevent immunopathology by inhibiting the ability of local neutrophils to produce tissue damaging TNFα and ROI. This PGE2-dependent process involves production of IL-10 by monocytes 40 and may go some way to explain earlier observations of enhanced neutrophil accumulation in the mucosa of *T. gondii* infected CCR2-deficient animals 125. These results support other reports that monocytes are not intrinsically inflammatory in nature, but are highly plastic cells that coordinate the first stages of tissue repair in response to damage, infection, or inflammation 130. Furthermore, the regulatory properties of elicited Ly6C^hi^ monocytes are diminished in GF mice, underlining how the local microenvironment determines the fate of monocytes in the mucosa 40.

Mϕ have also been implicated in Th2 cell-mediated resistance to intestinal helminth infections 131. Their numbers are expanded during infection by organisms such as *Trichuris muris*, *Nippostrongylus brasiliensis*, *Heligmosomoides polygyrus*, *Brugia malayi*, and *Litomosiodes sigmodontis* 132–134, and under these conditions, ‘alternative’ activation of mϕ by the Th2 products IL-4 and IL-13 leads to the production anti-parasitic mediators such as arginase and RELM-α 133,135. However, the exact role of mϕ in these infections is uncertain. The expulsion of these parasites is compromised in mice with mϕ depletion secondary to absence of CCL2 132 or administration of clodronate-containing liposomes 136,137. Alternatively activated mϕ are also partly responsible for the increased smooth muscle contractility which contributes to parasite expulsion 136. However, the protective effects of mϕ do not require them to produce arginase-1 138 or even to be polarized via the IL-4 receptor 139. Thus, mϕ may play an indirect role in protective immunity, perhaps by producing mediators that recruit other effector cells such as eosinophils and mast cells. Alternatively, they may be involved in the anti-inflammatory and tissue repair processes that are crucial components of the host response to multicellular pathogens and that are important properties of mediators such as arginase 140.

The origin of the alternatively activated macrophages in helminth infection is also uncertain. Although the requirement for CCL2 in parasite expulsion could indicate they are derived from recruited Ly6C^hi^ monocytes as in the models of inflammation discussed above, this has never been investigated directly. In addition, recent studies of macrophage expansion during helminth infection of the peritoneal and pleural cavities suggest that this may be driven by IL4- and CSF-1-dependent proliferation of the pre-existing resident population 141. The relative roles of *in situ* expansion of mϕ and monocyte recruitment during intestinal helminth infection could generate important insights into the plasticity and functional properties of mucosal macrophages.

## Monocytes and macrophages in human IBD

As discussed above, CD14^hi^ MP accumulate in the mucosa of IBD patients 12,24, and CD14^hi^ cells in human are the equivalent of Ly6C^hi^ monocytes in the mouse. As in mice, the CD14^hi^ cells that appear in human intestine display enhanced production of TNFα, IL-1β, and IL-6 123 and respiratory burst activity 46. They also retain their responsiveness to microbial products 24,122. Using the transfer of radiolabelled autologous blood monocytes, it has been demonstrated that the CD14^hi^ cells in the inflamed mucosa derive from circulating monocytes 142, likely in response to elevated levels of the monocyte chemoattractants CCL2 and CCL4 in the inflamed mucosa 39. In addition to their own pro-inflammatory effects, elicited monocytes in human have also been shown to orchestrate the recruitment of other innate effector cells, such as CCR3^+^ eosinophils through their production of CCL11 (eotaxin-1) 25,129. Furthermore, CD68^+^ mϕ in the inflamed mucosa have enhanced expression of CD40 143, which may facilitate their local interaction with effector T cells, analogous to elicited monocytes in mice. Thus, monocytes and mϕ appear to behave similarly in the inflamed intestine of humans and mice. Furthermore, our findings that analogous processes may govern mϕ differentiation in the two species intestine which seem to follow analogous processes in both steady state and in inflammation, suggest animal models remain a valuable means of exploring these processes.

## A role for elicited monocytes in T-cell priming?

An area of controversy in myeloid cell biology is whether monocytes can generate APCs that can contribute to the initiation of adaptive immune responses. Under the influence of IFNγ, monocytes in inflamed tissues acquire MHCII and are proposed to migrate to draining LN in a CCR7-dependent manner, where they may prime naive CD4^+^ T cells. By their ability to produce large amounts of cytokines, these monocyte-derived ‘DCs’ (mo-DCs) may complement and shape the generation of effector T cells by conventional, flt3L-dependent DCs 144. It has been suggested that Ly6C^hi^ monocytes entering the inflamed mucosa may behave in this manner, acquiring migratory capacity and transiting to the draining MLN 89,95. In support of this idea, Zigmond *et al*. 89 reported that a population of zbtb46^+^ CD11b^+^ Ly6C^−^ DCs in the colonic mucosa was depleted by administration of anti-CCR2 antibody and concluded that these were derived from Ly6C^hi^ CCR2^+^ monocytes 89. However, it is clear that some bona fide mature CD11b^+^ DCs can express CCR2 21,145, and pre-DC themselves may express CCR2 21, suggesting that *in vivo* depletion of CCR2^+^ cells may not be specific to monocytes and their progeny. Importantly, in our hands, CCR2^+^ CD11b^+^ DCs are derived from committed DC precursors and Ly6C^hi^ monocytes cannot repopulate any DC population in either steady state or inflamed intestine 12. Monocytes and mϕ also cannot be found in the pseudo-afferent intestinal lymph of mice with colitis (authors’ unpublished observations), and although monocyte-derived MHCII^+^ APC do accumulate in the MLN during experimental colitis, the vast majority of these arrive via the bloodstream in a CCR7-independent manner 16,144. Thus, while it seems likely that inflammatory monocytes may help shape effector T-cell responses in the mucosa itself, it remains unclear whether they can also act as migratory APCs and initiate T-cell responses in the MLN.

## Monocytes/macrophages during resolution of inflammation

Following the clearance of an infectious or inflammatory agent, the intestine must restore homeostasis so that chronic inflammation does not ensue. This is accompanied by major changes in the mϕ pool. During the resolution of colitis in mice, the expanded population of CX3CR1^int^ cells returns to normal size, paralleled by a reduction in granulocyte numbers 89. The mechanisms responsible for the contraction in inflammatory cells is unclear, although it seems likely that most elicited CX3CR1^int^ cells are cleared by apoptosis, as has been shown for the monocyte-derived cells recruited to the inflamed peritoneum 146. On the other hand, given that Ly6C^hi^ monocytes replenish CX3CR1^hi^ macrophages in the steady state, it is also conceivable that some of the elicited Ly6C^hi^ cells may convert into anti-inflammatory ‘resident’ mϕ once the inflammation begins to resolve, although this has not been shown directly. Even if this does not occur, we have already noted that the resident population of CX3CR1^hi^ mϕ appears to persist during mucosal inflammation and irrespective of their origin, these anti-inflammatory cells may play an active role in resolution of tissue damage (*Fig. *1). This is supported by the evidence discussed above that deletion of mϕ or MyD88 signaling in myeloid cells leads to increased susceptibility to experimental colitis and impaired tissue repair 42,43. Furthermore there is delayed resolution of DSS-induced colitis in CD68-dnTGFβRII mice which lack TGFβ signaling on mature mϕ 38. This is suggested to result in part from reduced IL-10 and enhanced IL-33 production by mucosal mϕ. The ability of anti-TNFα antibodies to promote mucosal healing in patients with IBD also correlates with the generation of ‘regulatory’ mϕ expressing the mannose receptor and able to suppress T-cell proliferation *in vitro* 147. Whether the promotion of tissue repair by mucosal mϕ reflects the mϕ-derived mediators, such as arginase-1 and PGE-2 that can influence mesenchyme function or epithelial stem cell renewal directly, or if downstream effects on other immune cells are involved remains to be determined.

## Concluding remarks

There have been significant advances recently in our understanding of how mϕ contribute to the maintenance of homeostasis in the intestine, largely due to improvements in methods for identifying them more precisely. Furthermore, we and others have established approaches which help explore the origins and developmental stages of intestinal mϕ. In particular, our findings show that the current paradigm that tissue-resident mϕ are derived from fetal precursors that self-renew *in situ*, does not apply to the intestine. Rather, the mucosal mϕ compartment requires continuous replenishment by blood monocytes that are Ly6C^hi^ in mice and CD14^hi^ in human and once considered to be inflammatory in nature. Under steady state conditions, these monocytes differentiate locally into anti-inflammatory mϕ that express scavenger receptors, MHCII, are highly phagocytic and hyporesponsive to pro-inflammatory stimuli, but produce large amounts of IL-10. This process is dictated by cues received from the immediate environment and ensures that mucosal mϕ can ingest and degrade dying tissue cells and any commensal bacteria that penetrate across the epithelial barrier without provoking inflammation. They can also help maintain epithelial integrity and contribute to physiological tissue remodeling. Interruption of the mechanisms that promote the development and/or function of anti-inflammatory mϕ leads to inflammation in response to luminal materials such as the microbiota. During inflammation, or in response to invading pathogens, the normal pattern of monocyte differentiation is disrupted, leading to the accumulation of potent pro-inflammatory effector monocyte/mϕ. Although these clearly play important roles in tissue pathology and protective immunity, they also may be needed for controlling harmful neutrophil activity and for generating the inert mϕ required for resolution of inflammation and tissue repair. These properties underline the plasticity of this population of ‘classical’ monocytes and understanding the local factors that determine their various fates after arrival in the mucosa under different conditions could hold promise as new therapeutic targets for the treatment of IBD.
